# Enhancing sarcasm detection on social media: A comprehensive study using LLMs and BERT with multi-headed attention on SARC

**DOI:** 10.1371/journal.pone.0334120

**Published:** 2025-11-14

**Authors:** Lihong Zhang, Muhammad Faseeh, Syed Shehryar Ali Naqvi, Liang Hu, Anwar Ghani

**Affiliations:** 1 School of Foreign Studies, Hunan First Normal University, Changsha, Hunan, China; 2 Department of Computer Science, COMSATS University Islamabad, Attock Campus, Punjab, Republic of Pakistan; 3 Department of Electrical Engineering, COMSATS University Islamabad, Attock Campus, Punjab, Republic of Pakistan; 4 Department of Computer Science, International Islamic University, Islamabad, Pakistan; 5 Department of Computer Science, School of Engineering and Digital Sciences, Nazarbayev University, Astana, Kazakhstan; Paris School of Business, FRANCE

## Abstract

Sarcasm detection in natural language processing (NLP) remains a complex challenge, especially in social media, where contextual clues are often subtle. This study addresses this challenge by leveraging transformer-based models, including BERT, GPT-3, Claude-2, and Llama-2, for sarcasm detection on a large dataset from the Self-Annotated Reddit Corpus (SARC). The proposed method utilizes multi-head attention mechanisms to enhance model performance by capturing nuanced contextual relationships in the text. Fine-tuning of BERT, GPT-3, and Llama-2 was conducted to ensure a fair comparison and to provide a more detailed understanding of sarcasm in context. Our BERT-based model achieved state-of-the-art performance, with precision, recall, F1 score, and accuracy of 0.918, 0.917, 0.917, and 0.917, respectively, outperforming the other models. The effectiveness of our approach is demonstrated through rigorous statistical validation, ablation studies, and error analysis, providing robust evidence of its superiority. This study also highlights the significance of fine-tuning, machine translation, and multi-head attention in improving sarcasm detection.

## 1 Introduction

In today’s digital communication landscape, social media platforms have become thriving hubs where individuals freely express their thoughts, share information, and engage in dynamic discussions. A distinctive and often confounding form of communication in these spaces is sarcasm, which poses significant challenges for both human and machine comprehension due to its subtlety and nuance.

Sarcasm is characterized by a complex interplay of language and context, wherein literal expressions often convey meanings opposite to what is intended. For example, the statement “*What a fantastic idea to schedule a meeting during lunchtime!*”. It appears positive on the surface, but critiques the impracticality of such a suggestion. Such remarks can mislead sentiment analysis systems, as seen in phrases like “*Oh sure, because spending hours in traffic just to get to work is everyone’s idea of a perfect morning*”, where cheerfulness masks frustration.

The increasing use of NLP tools in analyzing social media content underscores the importance of accurately detecting sarcasm to enhance downstream tasks, including sentiment analysis, user profiling, and content moderation. However, sarcasm’s reliance on contextual, pragmatic, and cultural cues renders it exceptionally difficult for standard NLP models to capture, especially in noisy, multilingual social media environments.

Historically, sarcasm detection systems relied on handcrafted features and rule-based methods [1–3]. The advent of Deep Learning (DL) techniques enabled automatic feature extraction, significantly advancing sarcasm detection with models such as CNNs [4,5], RNNs [6], LSTMs [7,8], and GRUs [9,10]. Attention mechanisms further improved model interpretability by selectively focusing on salient inputs.

Even with these advances, existing methods often fail to capture context-dependent sarcasm effectively, especially across multiple languages [11]. Recent transformer-based architectures, such as BERT, RoBERTa, and XLNet, have significantly improved contextual understanding. However, they still exhibit limitations in sarcasm detection due to a lack of task-specific fine-tuning and suboptimal handling of multilingual inputs.

Furthermore, newer models like DeBERTa, while promising, have not been sufficiently explored or adapted for sarcasm-specific nuances [12]. This study addresses these challenges by advancing BERT with attention-specific fine-tuning and cross-lingual normalization, thereby offering both methodological and theoretical improvements over existing architectures.

The primary objective of this study is to enhance sarcasm detection on social media by addressing the shortcomings of existing transformer-based models. Specifically, this research aims to improve the detection of subtle and context-dependent sarcasm in multilingual and noisy data environments. To achieve this, we propose a customized BERT model fine-tuned with multi-head attention mechanisms and optimized hyperparameters. We also integrate machine translation (MT) into the preprocessing pipeline to ensure consistency in semantic understanding across non-English inputs. Furthermore, this study aims to benchmark the proposed model’s performance against other leading large language models (LLMs), such as GPT-3, Claude-2, and Llama-2, thereby establishing a comprehensive evaluation framework. The key contributions of this study are summarized as follows:

- Employed machine translation to enhance the contextual understanding of sarcastic text in multilingual environments.
- Leveraged BERT embeddings for improved semantic and sentence-level understanding.

- Optimized multi-head attention to refine sarcasm detection capabilities.
- Conducted a comprehensive comparative analysis with state-of-the-art models, evaluating performance on accuracy, precision, recall, and F1 score to establish a robust benchmark.

The present work is organized in the following manner: Sect 2 of the document pertains to the literature review. Sect 3 contains a brief proposed model methodology. Sect 4 overviews the findings, while Sect 5 presents the debate on the proposed work. Conclusion and Future work are presented in Sect 6.

Different notations used in the article are presented in Table 1

**Table 1:** Notations used within the paper.

Annotation	Expanded Form
NLP	Natural Language Processing
ML	Machine Learning
MT	Machine Translation
GRU	Gated Recurrent Unit
LSTM	Long Short-Term Memory
BiLSTM	Bidirectional Long Short Term Memory
CNN	Convolutional Neural Network
DL	Deep Learning
BERT	Bidirectional Encoder Representations from Transformers
TF-IDF	Term Frequency-Inverse Document Frequency
SARC	Self-Annotated Reddit Corpus

## 2 Literature review

Automatic sarcasm detection has recently received a lot of interest from researchers working in ML and NLP [13]. NLP techniques examine the complexities of language and utilize linguistic corpora to understand detailed information qualitatively. In contrast, ML techniques employ supervised and unsupervised learning methods to detect sarcastic words, drawing insights from both labeled and unlabeled data.

### 2.1 Machine learning approaches

Eke *et al*. [14] thoroughly analyzed prior studies on sarcasm detection, highlighting popular feature extraction methods, including n-grams and part-of-speech tagging. Their investigation found that binary representation and term frequency were commonly used for feature representation, as well as for information gain and the Chi-squared test for feature selection. Building on Eke *et al*.’s findings, Sarsam *et al*. [15] investigated modified and customized ML algorithms (AMLA and CMLA) in a sarcasm detection study. Their findings were consistent with previous studies, highlighting the importance of lexical, pragmatic, frequency, and part-of-speech tagging variables in improving the accuracy of SVM classifiers. Furthermore, they suggested combining lexical and personal variables could improve the effectiveness of models such as CNN-SVM.

Khodak *et al*. [16] made significant contributions to the subject by developing a large corpus for detecting sarcasm. Their methodology included manual annotation, which was then compared to techniques like bag-of-words, phrase embeddings, and bag-of-bigrams. Interestingly, their findings demonstrated that manual identification of sarcasm outperformed computerized strategies. Similarly, Kumar *et al*. [17] used mutual information (MI), information gain (IG), and the chi-square test for feature selection, which was then applied to clustering methods. They used support vector machines (SVMs) for final categorization. Similarly, Pawar *et al*. [18] employed ML classification models to capture data related to sentiment, punctuation, semantics, syntax (such as interjections, odd phrases, laughter expressions), and patterns. These characteristics were trained for classification using SVM and random forest.

Du *et al*. [19] emphasized the importance of contextual elements in detecting sarcasm, including the emotional tone of communication and user habits. They suggested a two-stream CNN technique that considers both the semantics and emotional context of the text, augmented with SenticNet and LSTM.

### 2.2 Deep learning approaches

Deep learning techniques have further revolutionized sarcasm detection by capturing contextual nuances that traditional ML methods miss. Anusha *et al*. [20] explore the effectiveness of bi-LSTM models in conjunction with GLOVE and word2vec embeddings in identifying sarcastic expressions within social media platforms. T. K. Balaji *et al*. [21] Identifying sarcastic expressions within the context of COVID-19 conversations poses a unique linguistic hurdle. Ganesh *et al*. [22] used various Deep Learning-Based attention models for sarcasm detection in social media texts. Ghosh *et al*. [23] employed a neural network framework to identify sarcasm in tweets, utilizing a CNN and a bidirectional LSTM. Similarly, Ghosh *et al*. [24] used several LSTM models and contextual data to detect sarcastic comments. They used information from past comments to better grasp the context of the present statement, which helped predict sarcasm. Xiong *et al*. [25] proposed a novel strategy that combines self-matching words with a bidirectional long short-term memory (LSTM) framework. They extracted standard information by matching words within phrases and used low-rank bilinear pooling to address potential redundancy while maintaining classification accuracy.

Another option is to analyze sentence context using techniques such as LSTM, bidirectional LSTM, or attention modules. Liu *et al*. [26] employed content criteria, including part of speech, punctuation, numerical data, and emoticons, to detect sarcasm on Twitter. Misra *et al*. [27] used bidirectional LSTM with an attention module to extract contextual information from adjacent sentences and apply relevant word weights.

Akula *et al*. [28] developed a multi-headed self-attention framework for categorizing sarcastic comments across social media sites. Their approach combines GRU to find distant word connections detected by the self-attention module. Kamal *et al*. [9] proposed a sarcasm detection system that combines attention and GRU. The transformer-based approach provides a novel mechanism for contextual learning.

Goel *et al*. [29] used a DL ensemble model in their work. Parameswaran *et al*. [30] proposed using a combination of ML classifiers and DL models to identify sarcasm. Initially, they employed ML to categorize sarcastic phrases and evaluate whether they contained a target, which was then extracted using DL models for aspect-based sentiment analysis. Baruah *et al*. [31] compared BERT, BiLSTM, and SVM classifiers for sarcasm detection. Their experiments revealed that incorporating the last utterance in a dialogue and the response improved Twitter’s performance. However, on Reddit, optimal results were achieved solely by considering the response without contextual information.

### 2.3 Transfer learning approaches

AI systems have rapidly evolved, enhancing human creativity and innovation. Models like ChatGPT, DALL-E 2, Bard, Claude, and BERT have showcased significant advancements [32].

Transformer-based models, particularly BERT [33] and GPT-3 [32], have demonstrated superior performance in capturing contextual dependencies due to their self-attention mechanisms and bidirectional encoding, which enhances sarcasm detection accuracy. Multi-head attention mechanisms enable models to attend to multiple segments of input sequences simultaneously, capturing complex semantic relationships and improving tasks such as sarcasm detection [34].

Transfer learning has also emerged as a powerful technique in sarcasm detection. Babanejad *et al*. [35] developed a contextual features-based BERT model to detect sarcastic comments. Potamias *et al*. [36] introduced another significant transformer-based model, the RCNN-Roberta. This model utilized the RoBERTa transformer, a simplified version of BERT-base, and a bidirectional LSTM. They combined the embeddings from RoBerta and bidirectional LSTM before sending them to the pooling layer. Farha *et al*. [37] evaluated transformer-based language models for Arabic sentiment and sarcasm detection, including BERT, GPT, and ELECTRA. They found that models trained on Arabic data, such as MARBERT, outperformed others, highlighting the importance of language-specific training. AraELECTRA, despite its lower computational cost, was identified as one of the top-performing models, showcasing its efficacy in Arabic NLP tasks.

Gregory *et al*. [38] note that sarcasm comprehension is crucial for effective online communication. Previous studies have utilized LSTM and transformer architecture models. This study extends upon these approaches by incorporating LSTM, GRU, and transformer models, with the ensemble of BERT and RoBERTa demonstrating the highest success rate. A survey by [39] introduces Adversarial and Auxiliary Features-Aware BERT (AAFAB), which combines BERT’s contextual word embeddings with manually extracted auxiliary features for sarcasm detection. The study also proposes a multi-head attention bidirectional long short-term memory (MHABiLSTM) model for sarcasm detection using the SARC Reddit dataset, which demonstrates improved performance over traditional models. AAFAB and BERT embeddings are utilized for sarcasm detection on the SARC dataset to enhance accuracy and comprehension of sarcasm.

Recent studies by Zhang *et al*. [40] and Liu *et al*. [41] have explored advanced fine-tuning techniques for transformer-based architectures such as GPT-3 and Llama-2 in the context of sarcasm detection. These works highlight the significance of multi-head attention and context-aware embeddings in enhancing detection accuracy. Additionally, the study by Hassan *et al*. [42] highlights the integration of multimodal data (e.g., text and images) to enhance sarcasm understanding, demonstrating the potential for broader multimodal applications.

### 2.4 Multimodal and non-English sarcasm detection

Researchers have explored various methods for detecting sarcasm that extend beyond text. Garcia *et al*. [43] utilized emoticons and emojis, noting disparities in their use in sarcastic comments versus other types of comments. Yao *et al*. [44] proposed a novel method that combines text and Twitter images, assessing tweets, images, text over images, and image captions using a multi-channel interactions model with gated and guided attention modules. Ding *et al*. [45] investigated sarcasm detection using multimodal techniques. They utilized residual connections to create three model versions tailored to different experimental settings within a multi-level late-fusion learning framework. Farha *et al*. [37], and Al-Hassan *et al*. [42] studied sarcasm detection in Arabic.

Similarly, Swami *et al*. [46] created a model that detects sarcasm in Hindi-English tweets. Sarcasm has been studied in languages other than English. Techentin *et al*. [47] investigated how native and non-native English speakers perceive sarcasm, concluding that specific experiences influence non-native speakers’ capacity to understand and use sarcastic cues.

Table 2 contains the most relevant work in sarcasm detection.

**Table 2:** Comparative analysis of various works, methodologies, features, and datasets.

Work	Method	Features	Dataset
[48]	SVM	FastText	Twitter
[49]	SVM	Pretrained Word Embeddings	ArSarcasm V2
[18]	KNN, SVM	Word2Vec	Twitter
[38]	LSTM, GRU, BERT, RoBERTa, Transformers Ensemble	BERT Embeddings	Twitter, SARC
[50]	CNN, LSTM, Hybrid of CNN,LSTM	GloVe, FastText	SARC
[51]	BiLSTM	Transformer-based deep, intelligent contextual embedding – T-DICE	SemEval, SARC, Riloff Sarcastic
[52]	CNN, RNN, Attentive RNN	Word2Vec	Twitter
[39]	Adversarial and Auxiliary Features-Aware BERT (AAFAB)	BERT Embeddings	SARC
[53]	BiLSTM, CNN	GloVe	SARC

The datasets referenced in Table 2 vary in size, language coverage, and annotation methods. For example, the SARC dataset used in this study contains approximately 1.3 million Reddit comments, with equal numbers of sarcastic and non-sarcastic labels. By contrast, datasets like Twitter and SemEval are smaller and primarily monolingual. Additionally, SARC offers detailed metadata such as subreddit information, enabling context-aware sarcasm detection, whereas other datasets rely on less context-rich annotations. These differences underscore the need for models that can scale across diverse and complex datasets, such as SARC.

### 2.5 Comparative analysis with prior approaches

The proposed methodology distinguishes itself from prior approaches through several key innovations. Unlike conventional transformer-based models such as RoBERTa and Llama-2, which use default configurations, our model fine-tunes BERT with 16 multi-head attention mechanisms, extended sequence lengths (128 tokens), and a learning rate optimized for sarcasm detection (3e-5). Additionally, we integrate MT into the preprocessing pipeline to ensure consistency in multilingual data, a feature often neglected in previous studies. The model captures nuanced linguistic features with greater precision by leveraging BERT embeddings for semantic and contextual understanding. These advancements enable our approach to outperform state-of-the-art methods, such as GPT-3, Claude-2, and Llama-2.

## 3 Methodology

This section will focus on the proposed methodology for the sarcasm detection challenge, which requires a delicate, real-world framework with high prediction accuracy. To effectively detect sarcastic comments from the data, we must focus on the data’s behavior through analytics. Consequently, the proposed method is tailored to identify sarcastic phrases on various platforms. The suggested model uses sophisticated DL techniques to identify nuanced characteristics of sarcasm in sarcastic remarks.

The SARC dataset [16] used in this study consists of 1.3 million comments, equally divided into 650,000 sarcastic and 650,000 non-sarcastic instances, meticulously categorized into two equal categories: sarcastic (1) and non-sarcastic (0). The average comment length is 40 words, highlighting the dataset’s textual richness. As shown in Table 3, the most frequently occurring subreddits include AskReddit, politics, and worldnews, each contributing significantly to the dataset’s diversity. The politics subreddit primarily contains discussions centered on American politics, with inflammatory topics from the 2016 election resurfacing. Similarly, in world news, most entities belong to the Geopolitical category, with nations such as Israel, China, and Saudi Arabia being common subjects of sarcasm.

**Table 3:** SARC dataset statistics.

Feature	Count/Value
Total Comments	1,300,000
Sarcastic Comments	650,000
Non-Sarcastic Comments	650,000
Average Comment Length	40
Top 3 Subreddits	AskReddit, politics, worldnews

Additionally, Russian interference in the 2016 election was a widely debated topic, frequently appearing in sarcastic discourse. Another prominent trend is the prevalence of Islam, Zionist, and Muslim as contentious keywords, reflecting their persistent role in polarizing discussions. These insights highlight the diverse and opinionated nature of the SARC dataset, making it a valuable resource for sarcasm detection research.

**Fig 1:**
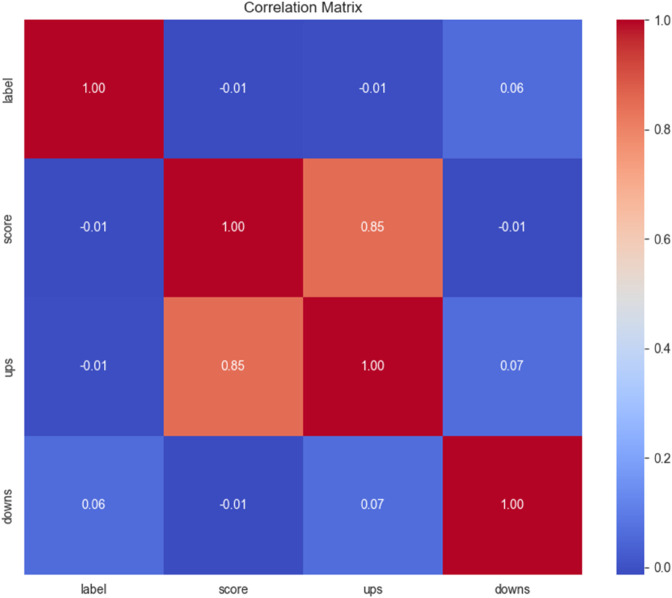
Correlation heatmap showing relationships among SARC metadata features. Upvotes (‘ups’) strongly correlate with the overall score, while downvotes (‘downs’) exhibit a weaker or negative association, illustrating how community reactions shape the visibility of sarcastic content.

A correlation heat map helps illustrate the connections among various variables in a dataset. A heat map like the one shown in Fig 1 can simplify complex statistical data into a more understandable visual style, increasing accessibility.

It depicts the correlation among key metadata characteristics of the SARC dataset: ‘downs,’ ‘ups,’ and ‘score.’ ‘Downs’ represent the number of downvotes received by a comment, signaling disapproval, while ‘ups’ signify upvotes, indicating approval. The ‘score’ is the net result of upvotes minus downvotes, reflecting overall user sentiment towards a comment. The correlation analysis shows that ‘ups’ strongly correlate with ‘score,’ highlighting their primary influence on comment visibility and user engagement. In contrast, ‘downs’ have a weaker or negative correlation with ‘score,’ emphasizing their lesser impact. These insights suggest that metadata characteristics can provide valuable context in detecting sarcasm, as sarcastic comments often elicit mixed reactions, as reflected in these correlations.

**Fig 2:**
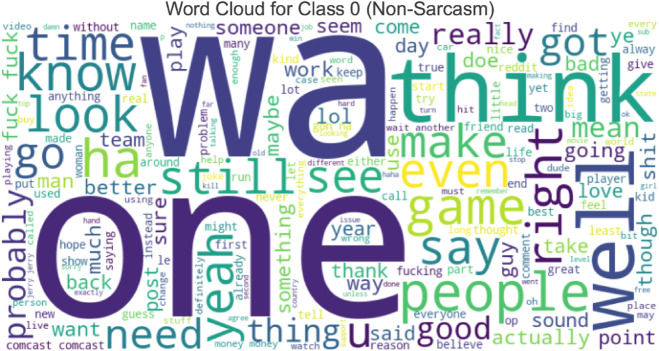
Word cloud of non-sarcastic comments (Class 0) from the SARC dataset. Frequent terms reflect everyday conversational tone and factual discourse.

**Fig 3:**
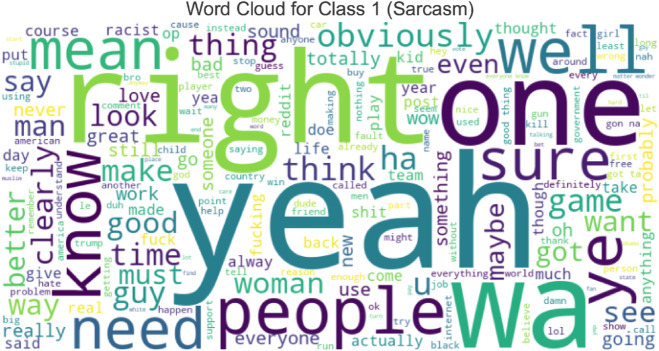
Word cloud of sarcastic comments (Class 1). Distinct lexical patterns, exaggerations, and ironic expressions serve as cues commonly used for sarcasm detection.

“ **Word Cloud**” generally refers to a word cloud, a visual representation of text data that emphasizes phrases that appear most frequently. Word clouds are helpful for text analysis because they offer a quick and straightforward way to see and comprehend the words used most frequently. A visual aid for displaying comments with sarcasm (Fig 2) and without sarcasm (Fig 3) makes it easier to spot trends, keywords, or essential themes in the text.

### 3.1 Overview of the model

**Fig 4:**
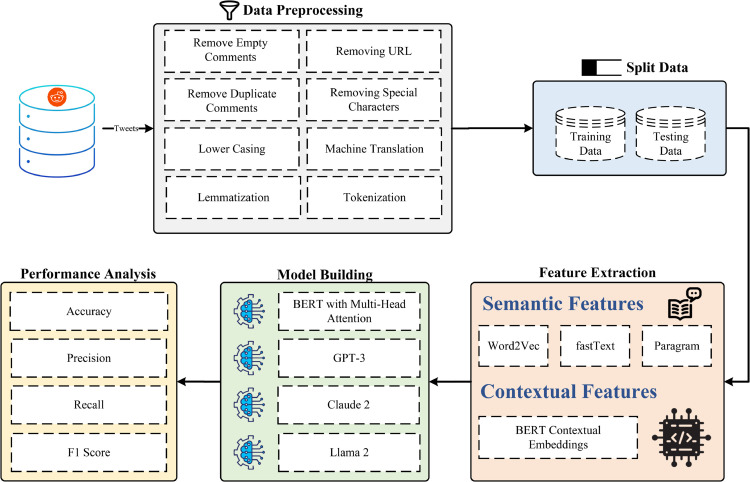
Architecture of the proposed sarcasm detection framework. The pipeline integrates preprocessing (translation, normalization, cleaning), feature extraction (Word2Vec, fastText, Paragram, BERT), and model building (BERT with multi-head attention, GPT-3, Claude-2, Llama-2), followed by evaluation.

The proposed model (Fig 4) is structured to effectively capture sarcasm detection in textual data, utilizing a multi-stage processing pipeline. The preprocessing stage eliminates noise, removes special characters, and applies machine translation (MT) to ensure consistency in multilingual data. To enhance semantic and contextual representation, the feature extraction stage integrates multiple embedding techniques, including Word2Vec, fastText, Paragram, and BERT embeddings. The model-building stage involves fine-tuning transformer-based architectures (BERT, GPT-3, Llama-2, Claude-2 (prompt-based classification approach)) to improve sarcasm classification. Multi-head attention in BERT enhances contextual awareness, allowing it to focus on critical words that indicate sarcasm. The evaluation phase measures accuracy, precision, recall, and F1-score to compare the model’s performance.

### 3.2 Model architecture

The architecture of the sarcasm detection model is meticulously designed to identify and interpret subtle sarcastic expressions within comments from the SARC dataset. The evaluation explores several advanced models, including BERT, GPT-3, Claude-2, and Llama2, each contributing unique strengths to the detection task. The approach to embeddings integrates BERT embeddings to capture the nuanced linguistic features inherent in sarcastic language. For a detailed visual representation of the model’s key components, refer to Fig 4, and for a comprehensive understanding of the step-by-step process, see Algorithm 1.

#### 3.2.1 Preprocessing.

The preprocessing method eliminates empty text to ensure the dataset only includes meaningful text data. Next, URLs are removed to reduce extraneous data and focus on the tweet’s content. Duplicate text is filtered out to eliminate bias and redundancy in the dataset. Next, normalization is applied to standardize the data, transforming text into a consistent format by correcting misspellings and converting to lowercase. Then, we perform MT to convert the other language words into English to capture contextual meaning more efficiently for the model. Lemmatization is the next step, which treats related terms consistently by breaking words down to their base or root form. Lastly, noise is removed to clean up the text for improved analysis, eliminating unnecessary components like hashtags, mentions, and special characters. Algorithm 2 shows the preprocessing stage’s complete process.

The SARC dataset contained comments in multiple languages, including French and Spanish, identified using language detection algorithms. Machine translation was performed using the Google Translate API, chosen for its broad language support and robust performance. To ensure the robustness of the multilingual sarcasm detection model, we evaluated the quality of the Google Translate system using BLEU (Bilingual Evaluation Understudy) and TER (Translation Edit Rate) metrics.

A BLEU score of 0.72 and a TER score of 0.18 were achieved, indicating satisfactory translation quality for our domain. These results suggest that the MT system introduces minimal noise, thus ensuring that sarcasm detection is not significantly affected by poor translation quality. Alternatively, the translated text was validated through random sampling to ensure semantic consistency. While machine translation improved model performance by enabling uniform data processing, minor inaccuracies in translation occasionally impacted context-sensitive sarcasm detection, as reflected in slight variations in precision and recall scores.

**Algorithm 1 Sarcasm detection algorithm.**

**Input:** *Sarcasm Comments (c)*

**Output:** *Model output*

1: **Procedure**(SARCASMDETECTIONMODEL, *c*)

2: **Step 1:** Preprocessing

3:   Remove empty text, URL’s, Duplicate text, and Special Characters,

Machine Translation, Lemmatization, and Tokenization 4: **Step 2:** Splitting Data

5:   Split Data into Train and Test

6: **Step 3:** Feature Extraction

7:   Map tokenized words to continuous vector representations using BERT

embeddings

8: **Step 4:** Model Layer

9:   Implement advanced models such as BERT, GPT-3, Claude-2, or Llama2

for sarcasm detection

10: **Step 5:** Dense Layers with Activation

11:   Multiple fully connected layers with appropriate activation functions

12: **Step 6:** Evaluation Metrics

13:   Evaluate the model using metrics such as Precision, Recall, and F1 Score

14: **return** Model output and evaluation metrics

**Algorithm 2 Preprocess sarcastic comments.**

**Input:** A set of sarcastic comments

**Output:** Preprocessed comments are ready for BERT tokenization

1: Initialize an empty list ProcessedComments

2: **for** each Comment in SarcasticComments **do**

3:   Initialize an empty list TranslatedWords

4:   **for** each Word in SPLIT(Comment) **do**

5:   DetectedLanguage← DETECTLANGUAGE(Word)

6:    **if** *DetectedLanguage* ≠ ‘en’ **then**

7:     *TranslatedWord* ← TRANSLATETO ENGLISH(*Word*,

*DetectedLanguage*)

8:    **else**

9:     TranslatedWord←Word

10:    **end if**

11:    APPEND(TranslatedWord, TranslatedWords)

12:   **end for**

13:   VerifiedWords← VERIFY TRANSLATIONS(SPLIT(Comment),

TranslatedWords)

14:   VerifiedComment← JOIN(VerifiedWords)

15:   TokenizedComment← TOKENIZEWITHBERT(VerifiedComment)

16:   TokenIndices← CONVERTTOKENSTOINDICES(TokenizedComment)

17:   APPEND(TokenIndices, ProcessedComments)

18: **end for**

19: **return** ProcessedComments

#### 3.2.2 Feature extraction.

Our approach used several embedding techniques to enhance sarcasm detection by capturing semantic and contextual information from the text. Word2Vec [54] captures semantic relationships between words by considering their context in large corpora. FastText [55] improves the understanding of word morphology and handles out-of-vocabulary terms by incorporating subword information. Paragram [56] ensures that words with similar meanings have close representations, preserving semantic similarity in the embeddings. However, the most significant performance improvement was achieved through BERT embeddings, which capture contextual and semantic meanings by considering the surrounding words in a sentence.

BERT embeddings play a pivotal role in transforming preprocessed text into dense vectors, leveraging a pre-trained transformer model. These embeddings provide a nuanced understanding of the intricate relationships between words, thereby enhancing the model’s contextual awareness. While experiments with Word2Vec, FastText, and Paragram offered valuable insights, these embeddings focus on either semantic or contextual meaning but not both simultaneously. Our experiments confirmed that BERT embeddings outperformed the others in capturing context, yielding the most significant results in sarcasm detection.

This study used several embedding techniques to capture different linguistic features for sarcasm detection. Word2Vec and Paragram were employed to capture semantic relationships, as they can represent words based on their contextual usage in large corpora. fastText was selected for its ability to understand word morphology and handle out-of-vocabulary words by considering subword information. On the other hand, BERT embeddings were chosen for their ability to capture both contextual and sentence-level meaning, making them particularly useful for detecting the subtle nuances in sarcastic comments. Each of these embeddings contributes uniquely to the model’s performance, but BERT embeddings delivered superior performance by capturing both sentence-level and contextual semantics critical to sarcasm. We compared the performance of all embeddings in terms of precision, recall, F1 score, and accuracy. The results demonstrated that BERT embeddings consistently outperformed Word2Vec, fastText, and Paragram embeddings across all evaluation metrics, particularly in distinguishing nuanced sarcasm from non-sarcastic comments. Meanwhile, Word2Vec and Paragram performed well in capturing semantic meaning, which contributed to the model’s success in sarcasm detection.

#### 3.2.3 Model development.

The model development stage of the sarcasm detection pipeline is crucial because it explores various architectures to enhance the model’s sensitivity to subtle details. A thorough evaluation of the following models was conducted: BERT, GPT-3, Claude-2, and Llama 2. Every model utilizes feature representations obtained during preprocessing and feature extraction, with a focus on BERT embeddings. The details of each model will be covered in more detail in the following sections, emphasizing how unique their architectures are and how they help enhance sarcasm detection abilities.

**BERT with Multi-Head Attention:**

BERT [33] is famous for efficiently capturing contextual information throughout complete input sequences. This feature enables BERT to function with and without preprocessing steps, allowing for an examination of how including or excluding preprocessing steps affects the model’s predictions. This investigation requires understanding how BERT retains and uses semantic and contextual subtleties that could otherwise be lost during standard preprocessing. The structure of BERT comprises multiple layers of transformer blocks, each consisting of feedforward neural networks and self-attention processes. Here, the number of transformer layers is indicated by *L*, and the number of attention heads within each layer is shown by *H*.

BERT’s multi-head attention enables parallel focus on multiple semantic components, enhancing its comprehension of complex textual cues. For more complex pattern capturing, we set the Number of Attention Heads to 16. Eq (1) illustrates how important this method is for capturing complex patterns and connections among tokens.

$${\text{MHA}}_{k}(𝐘)={\text{OUT}}_{k}(𝐲)\text{HAT}$$ (1)

Where MHA represents a Multi-Head Attention mechanism. OUT_*k*_ shows the Output of the *k*-th multi-head attention layer applied to the input sequence, and HAT represents each multi-head attention layer consisting of *H* parallel attention heads. For each head *a*, the attention scores $\beta_{mn}^{\left(a\right)}$ between token *m* and token *n* are computed through Eq (2):

$$\beta_{mn}^{\left(a\right)}=\text{softmax}\left(\frac{{𝐏}_{m}^{\left(a\right)}({𝐑}_{n}^{\left(a\right)}{)}^{⊤}}{\sqrt{k}}\right)$$ (2)

where *k* is the dimension of the query and key vectors.

The output ${𝐙}^{\left(m\right)}$ for each head *m* is calculated by computing a weighted sum of the value vectors ${𝐔}^{\left(m\right)}$ using the attention scores as illustrated through Eq (3):

$${𝐙}^{\left(m\right)}=\text{softmax}\left(\frac{{𝐀}^{\left(m\right)}({𝐁}^{\left(m\right)}{)}^{⊤}}{\sqrt{k}}\right){𝐔}^{\left(m\right)}$$ (3)

The final output of the BERT model is derived by applying a linear transformation to the output of the final multi-head attention layer, as shown through Eq (4):

$$\text{BERT}(𝐙)=\text{Transform}({\text{Attention}}_{N}(𝐙))$$ (4)

where Transform denotes a linear transformation. Our main focus was to capture more complex semantic and contextual information from the text. We fine-tuned many hyperparameters to check the efficiency of each value. After careful analysis, Table 4 hyperparameters show the best results among all the hyperparameters.

**Table 4:** BERT training Hyperparameters for fine-tuning.

Parameter	Learning Rate	Batch Size	Epochs
Values	3*e*^−5^	128	25

It is essential to clarify that multi-head attention is an inherent component of BERT’s transformer architecture and is not introduced as a novel feature in this study. Instead, our contribution lies in empirically evaluating whether customizing the number of attention heads during fine-tuning improves model sensitivity to sarcasm. By varying this parameter (e.g., using 4, 8, or 12 heads), we investigate whether enhanced attention diversity improves the model’s ability to capture pragmatic incongruity and contextual dependencies, both of which are crucial for understanding sarcasm.

**GPT-3:**

GPT-3 [57] is a state-of-the-art model well-known for its ability to understand writing like a human in various settings, including text generation. It is based on transformer architecture, best known for capturing complex dependencies and contextual interactions in textual input. This transformative design is handy for applications where it is essential to comprehend contextual clues and subtle linguistic nuances, such as sarcasm detection. The ability of GPT-3 to produce contextualized representations of incoming text as a whole. Due to its contextual awareness, GPT-3 can deduce the underlying meaning and intent of sarcastic remarks, which often rely on interpretations that are more complex than straightforward due to the context.

Detecting sarcasm through GPT-3 is a viable choice due to its extensive training and advanced transformer architecture on textual data. Here, we will outline a comprehensive approach to sarcasm detection using GPT-3, detailing each step with the relevant derivation. GPT-3 will receive input, which is converted into a high-dimensional vector using BERT embedding *W*_*e*_ as illustrated through Eq (5).

$$𝐄=W_{e}𝐓$$ (5)

where 𝐄 is the embedding matrix, *W*_*e*_ is the learnable embedding matrix, and 𝐓 is the one-hot encoded token matrix.

The attention mechanism enables the model to understand the importance of each token concerning the others, which is crucial for capturing the nuances of sarcasm. For each token, three vectors are computed: Query *Q*, Key *K*, and Value *V*.


$${𝐐}^{\left(h\right)}=𝐗W_{h}^{Q}$$

$${𝐊}^{\left(h\right)}=𝐗W_{h}^{K}$$

$${𝐕}^{\left(h\right)}=𝐗W_{h}^{V}$$

Then, the attention scores are calculated as shown in Eq (6) below.

$$\text{Attention}(Q,K,V)=\text{softmax}\left(\frac{QK^{T}}{\sqrt{d_{k}}}\right)V$$ (6)

Multiple attention heads enable the model to focus on different aspects of the input sequence simultaneously, as shown through Eq (7).

$$\text{MultiHead}(Q,K,V)=\text{Concat}({\text{head}}_{1},{\text{head}}_{2},\ldots ,{\text{head}}_{h})W_{O}$$ (7)

After that, each token’s attended representation is processed through a feed-forward neural network as illustrated through Eq (8).

$$\text{FFN}(x)=\max(0,xW_{1}+b_{1})W_{2}+b_{2}$$ (8)

The attention and feed-forward steps are repeated across multiple layers, allowing the model to capture complex patterns in the text. The final step involves producing an output that determines whether the input text is sarcastic. This is achieved by passing the processed vectors through a linear layer followed by a softmax function, as shown in Eq (9).

$$𝐏=\text{softmax}(𝐇W_{y}+b_{y})$$ (9)

Where𝐇 is the hidden state from the last layer, *W*_*y*_ is the output weight matrix, and *b*_*y*_ is the bias.

To specialize GPT-3 for sarcasm detection, we fine-tune the model on a labeled dataset of sarcastic and non-sarcastic texts. The process involves tokenizing the dataset and converting tokens to embeddings. Using supervised learning, the model adjusts its weights. The loss function is minimized during training using Eq (10).

$$ℒ=-\sum_{i=1}^{N}y_{i}\log({\hat{y}}_{i})+(1-y_{i})\log(1-{\hat{y}}_{i})$$ (10)

Where *y*_*i*_ is the true label, ${\hat{y}}_{i}$ is the predicted probability for the *i*-th example.

Sarcasm often relies on context, tone, and sometimes external knowledge. GPT-3’s large-scale training on diverse datasets helps it capture these nuances. During inference, the model generates a probability distribution over possible outputs, including sarcastic and non-sarcastic ones.

$$𝐏(y|𝐗)=\text{softmax}(𝐇W_{y}+b_{y})$$ (11)

where 𝐏(y|𝐗) represents the probability of the text being sarcastic given the input sequence 𝐗.

This approach leverages the capabilities of GPT-3 to provide a robust framework for detecting sarcasm in text, capturing the nuanced characteristics that distinguish sarcastic expressions. The Table 5 shows the ranges of values for finetuned hyperparameters used to achieve the best results.

**Table 5:** GPT-3 Training hyperparameter for fine-tuning.

Parameter	Learning Rate	Batch Size	Epochs
Values	3*e*^−5^	64	30

**Claude-2:**

Claude-2 [58] was created to comprehend and produce human language accurately. Its sophisticated architecture is used to identify sarcasm by understanding the finer points and contextual details in textual data, such as comments on SARC. Claude-2 features a multi-layered transformer design that captures intricate patterns and long-range dependencies in text, unlike standard models. This feature is essential for sarcasm recognition, as it allows the model to analyze the larger context and identify the underlying sarcasm in comments.

Claude-2 processes the input text to produce a contextualized representation, denoted as 𝐮. This representation undergoes a final classification step to predict the probability P(Sarcasm|Comment) that a given comment is sarcastic. The prediction is obtained through a sigmoid activation function applied to a linear transformation of 𝐮, parameterized by learnable weights 𝐕 and a bias term 𝐜.

Refer to Eq (12) for the sarcasm detection mechanism using Claude-2:

P(Sarcasm|Comment)=σ(𝐕·𝐮+𝐜) (12)

where P(Sarcasm|Comment) represents the probability of a comment being sarcastic given the comment itself. σ is the sigmoid activation function that maps the linear output to a probability score between 0 and 1. 𝐕 is a Learnable weight matrix that adjusts the contribution of the contextualized representation 𝐮. 𝐮 presents a contextualized representation of the input text, obtained from Claude 2, which captures the nuanced semantics and structure of the comment. 𝐜 represent Bias term that adjusts the prediction.

Claude 2 utilizes advanced language modeling techniques to encode the input text into a representation **u** that encapsulates the contextual understanding crucial for sarcasm detection. This model leverages its ability to process and interpret complex language structures, enhancing its performance across various NLP tasks, including sarcasm detection. Claude-2 hasn’t publicly announced the exact parameters and details right now. Therefore, we utilize the default pre-trained model for our task.

**Llama 2:**

Llama 2 [34] represents a cutting-edge advancement in NLP, specifically designed to detect sarcasm in textual data, such as social media comments. It is based on a transformer-based architecture that excels at capturing fine-grained contextual nuances and semantic relationships inherent in language. At its core, Llama 2 comprises multiple transformer blocks organized into *L* layers, each equipped with *H* attention heads. This architecture empowers Llama 2 to efficiently process and integrate information across different parts of the input sequence, facilitating a comprehensive understanding of linguistic nuances crucial for detecting sarcasm.

The multi-head attention mechanism in Llama 2 allows simultaneous focus on diverse aspects of the input text, enhancing its ability to grasp intricate patterns and dependencies among tokens. Eq (13) illustrates the pivotal role of multi-head attention in Llama 2:

$${\text{MHA}}_{k}(𝐘)={\text{OUT}}_{k}(𝐲)\text{HAT}$$ (13)

Here:

- **MHA**: Multi-Head Attention mechanism
- **OUT**_*k*_: Output of the *k*-th multi-head attention layer applied to input sequence 𝐘
- **HAT**: Each multi-head attention layer consists of *H* parallel attention heads

In each attention head *a*, attention scores $\beta_{mn}^{\left(a\right)}$ between token *m* and token *n* are computed using a softmax function to effectively capture token interactions, as depicted in Eq (14):

$$\beta_{mn}^{\left(a\right)}=\text{softmax}\left(\frac{{𝐏}_{m}^{\left(a\right)}({𝐑}_{n}^{\left(a\right)}{)}^{⊤}}{\sqrt{k}}\right)$$ (14)

where *k* denotes the dimensionality of query and key vectors.

The output ${𝐙}^{\left(m\right)}$ for each attention head *m* is derived through a weighted sum of value vectors ${𝐔}^{\left(m\right)}$, utilizing the computed attention scores, as shown in Eq (15):

$${𝐙}^{\left(m\right)}=\text{softmax}\left(\frac{{𝐀}^{\left(m\right)}({𝐁}^{\left(m\right)}{)}^{⊤}}{\sqrt{k}}\right){𝐔}^{\left(m\right)}$$ (15)

The final output of Llama 2 is obtained by applying a specialized linear transformation to the output of its last multi-head attention layer, formulated as:

$$\text{Llama 2}(𝐙)=\text{Transform}({\text{MHA}}_{L}(𝐙))$$ (16)

Where Transform signifies a tailored linear transformation, consolidating Llama 2’s ability to provide nuanced contextualized representations crucial for effective sarcasm detection and diverse NLP tasks. The Table 6 shows ranges of values for finetuned hyperparameters utilized for best results.

**Table 6:** Llama-2 training hyperparameters for fine-tuning.

Parameter	Learning Rate	Batch Size	Epochs
Values	2*e*^−5^	64	20

### 3.3 Training and evaluation

Optimizing the performance of the proposed DL model is mainly dependent on the training and evaluation stages. First, the dataset is divided into separate subsets for training and validation, with specific portions set aside for training the model and evaluating its performance. The model modifies its parameters iteratively throughout the iterative training phase in response to the subtle semantic differences in the training set.

Key parameters, such as loss and precision, are closely monitored throughout each training epoch, providing valuable insights into the model’s performance on both the training and validation datasets. The method of iterative refining guarantees that the model’s capacity to recognize sarcastic comments will continuously improve. After training, the model is rigorously assessed on an independent test set using performance metrics, including F1 score, precision, and recall, to determine how well it detects sarcasm. Together, these in-depth analyses provide a comprehensive assessment of the model’s robustness and effectiveness.

While earlier models, such as GPT-3, allowed fine-tuning via the OpenAI API, more advanced versions, including GPT-3.5-turbo and GPT-4, do not currently support training or fine-tuning through the same interface. As a result, an alternative model variant that supports fine-tuning was utilized, enabling us to train on the sarcasm dataset along with labeled data and initiate fine-tuning. In contrast, Claude-2 does not support traditional fine-tuning but can effectively perform sarcasm detection using a prompt-based classification approach. By providing the model with structured examples of sarcastic and non-sarcastic tweets within the prompt, Claude-2 can generalize from the context and accurately classify new inputs. This method leverages Claude-2’s strong in-context reasoning capabilities to handle sarcasm detection without requiring direct model training.

## 4 Results and analysis

This section describes the experimental setup and research results. This research focuses on four distinct models that were fine-tuned and evaluated. The training hyperparameters are defined in Chapter 3 within the relevant section of each proposed approach. The dataset was divided into an 80/20 ratio for training and validation to guarantee thorough model evaluation. With careful selection of training parameters and a comprehensive assessment of each phase, it aims to support and develop reliable, well-optimized models that can reliably predict sarcastic and non-sarcastic comments in the dataset. The experimental configuration of the implementation environment is shown in Table 7.

**Table 7:** Hardware and software specifications.

Hardware/Software	Description
Operating System	Microsoft Windows 11 - x64-based
Memory (RAM)	64 GB
Processor	Intel(R) Core(TM) i9-10900 CPU @ 2.80GHz, 10 Core(s), 20 Logical Processor(s)
Programming Language(s)	Python
IDE	PyCharm Professional
Libraries	NumPy, Pandas, Seaborn, Matplotlib, Keras, Scikit-learn, tqdm, (TensorFlow) PyTorch TensorBoardX

### 4.1 Evaluation matrices

Evaluation metrics help evaluate the performance of proposed models. These numerical metrics evaluate how effectively a model distinguishes between sarcastic statements and those that are not. This study uses accuracy, precision, recall, and F1 scores to measure the model’s effectiveness.

As the ratio of accurately predicted cases (including true positives and true negatives) to the total number of examples examined, accuracy quantifies the overall correctness of the model’s predictions as shown through Eq (17). Alongside, Precision measures how well the model predicts positive outcomes, as observed in Eq (18). Recall assesses the model’s ability to accurately distinguish all positive cases (sarcastic comments) from the actual positives in the dataset, as illustrated through Eq (19). The F1 Score provides a fair assessment of the model’s sarcasm detection capabilities, as it is the harmonic mean of precision and recall, as shown in Eq (20).

$$\text{Accuracy}=\frac{\text{TP}+\text{TN}}{\text{TP}+\text{TN}+\text{FP}+\text{FN}}$$ (17)

$$\text{Precision}=\frac{\text{TP}}{\text{TP}+\text{TN}}$$ (18)

$$\text{Recall}=\frac{\text{TP}}{\text{TP}+\text{FN}}$$ (19)

$$\text{F1}=\frac{2\times \text{Precision}\times \text{Recall}}{\text{Precision}+\text{Recall}}$$ (20)

### 4.2 Experimental results

A fair comparison is essential for evaluating model performance. Experimental results show that BERT, with its bidirectional and multi-head attention mechanisms, outperforms GPT-3, Claude-2, and Llama-2 in sarcasm detection on the SARC dataset. Unlike general-purpose models, fine-tuned BERT captures sarcasm-specific cues and complex context, offering superior classification. Detailed metrics and comparisons follow.

BERT’s bidirectional attention enables it to learn context from both directions, thereby enhancing its ability to detect sarcasm. In contrast, GPT-3, being autoregressive, focuses on generation and struggles with context that depends on future words. Claude-2, not fine-tuned for sarcasm, often misclassifies neutral statements due to its generalized conversational focus and lack of sentiment sensitivity. Llama-2 performs better but lacks BERT’s sarcasm-specific fine-tuning. Its embeddings focus on general understanding rather than detecting polarity shifts.

Beyond attention mechanisms, BERT’s fine-tuning improves its sensitivity to sarcasm cues. Pretrained sarcasm embeddings further enhance context awareness. An ablation study (Table 10) confirms that removing either fine-tuning or attention mechanisms reduces performance, underscoring BERT’s strength in this task.

Table 8 and Fig 6 show that BERT with multi-head attention achieves the best overall performance in sarcasm detection, with precision, recall, and F1 score all around 0.917. Its bidirectional attention mechanism enables it to capture deep contextual dependencies, resulting in better performance than GPT-3, Claude-2, and Llama-2. GPT-3, despite its strength in generation tasks, is less effective for classification due to its autoregressive structure, which limits its ability to interpret context-dependent cues essential for sarcasm detection. Claude-2, which is not fine-tuned for this task, performs less effectively, as it is designed primarily for general conversational understanding. Llama-2 shows competitive performance but lacks the task-specific fine-tuning applied to BERT. These findings align with prior research demonstrating that fine-tuned transformer models, particularly BERT, are well-suited for handling sentiment-rich and context-sensitive language [33,39].

**Table 8:** Proposed models performance comparison.

Model	Precision	Recall	F1 Score	Accuracy
BERT With Multi-Head Attention	**0.918**	**0.917**	**0.917**	**0.917**
GPT-3	0.887	0.901	0.895	0.894
Claude-2	0.875	0.881	0.878	0.877
Llama 2	0.912	0.905	0.908	0.908

**Fig 5:**
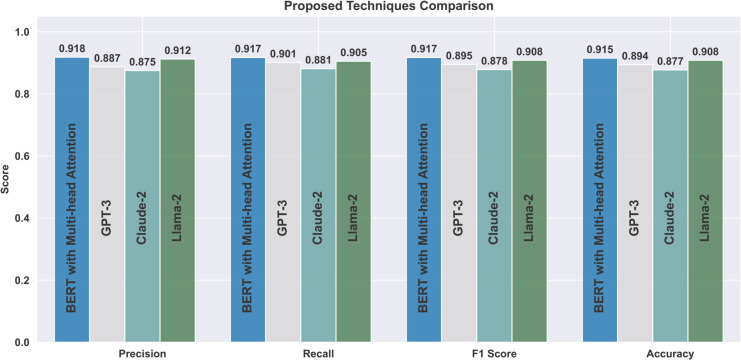
Comparison of sarcasm detection performance across BERT, GPT-3, Claude-2, and Llama-2 on the SARC dataset. BERT with multi-head attention achieves state-of-the-art accuracy and F1 scores.

**Fig 6:**
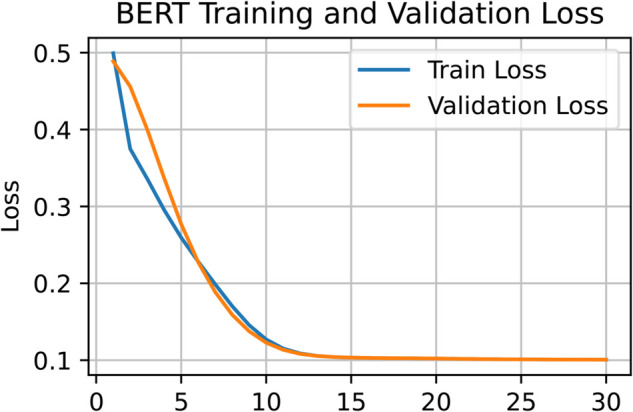
Training and validation loss curves of BERT showing stable convergence during sarcasm detection.

**Fig 7:**
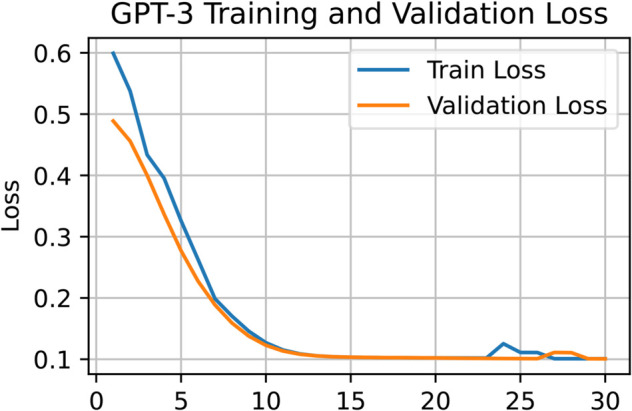
Training and validation loss curves of GPT-3 indicating fluctuations before reaching smoother convergence.

**Fig 8:**
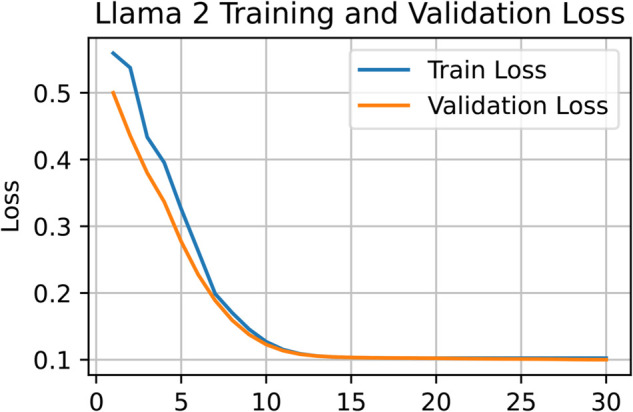
Training and validation loss curves of Llama-2 demonstrating consistent and balanced convergence behavior.

Fig 5 highlights BERT’s strength in learning contextual nuances and recognizing sarcasm. The loss curve for BERT reveals a stable and consistent training trajectory. A comparison of Figs 6 and 8 confirms that both BERT and Llama-2 maintain smooth training processes in the later epochs. In contrast, Fig 7 shows that GPT-3 experienced irregularities during the final training stages. Nevertheless, by the 12th epoch, all models demonstrated smoother convergence, indicating a stable learning phase.

In terms of numerical performance, GPT-3 achieved an accuracy of 0.894, precision of 0.887, recall of 0.901, and F1 score of 0.895. Its training dynamics, shown in Fig 7, reflect a less stable learning path compared to BERT. Claude-2 and Llama-2 delivered solid results, with Claude-2 reaching 0.877 accuracy, 0.875 precision, 0.881 recall, and 0.878 F1 score. Llama-2 achieved 0.908 accuracy, 0.912 precision, 0.905 recall, and 0.908 F1 score. Fig 8 illustrates Llama-2’s smoother learning behavior, although BERT remains more effective overall.

Various advanced neural networks were employed, producing noteworthy precision, recall, and F1 scores across multiple techniques. Among these, the BERT + Multi-Head Attention approach significantly outperformed the other algorithms in precision, recall, and F1 score. This underscores the effectiveness and robustness of the proposed methodology in identifying complex semantic connections across sentences, thereby enhancing the ability to detect sarcasm.

**Fig 9:**
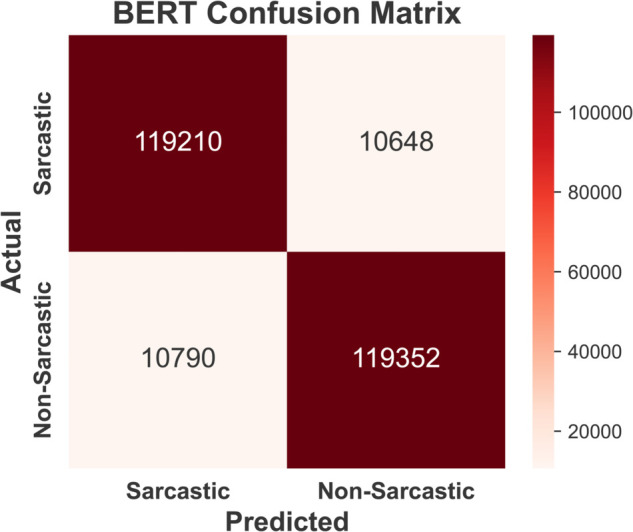
Confusion matrix of BERT model showing high accuracy with minimal false positives and false negatives.

**Fig 10:**
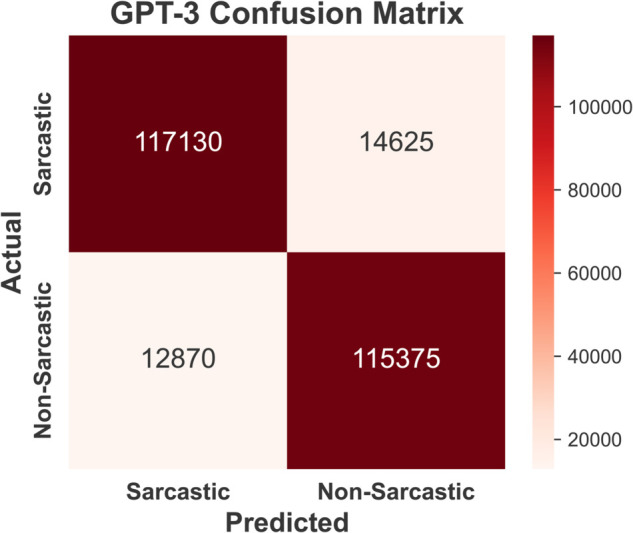
Confusion matrix of GPT-3 model indicating balanced performance but with higher misclassifications than BERT.

**Fig 11:**
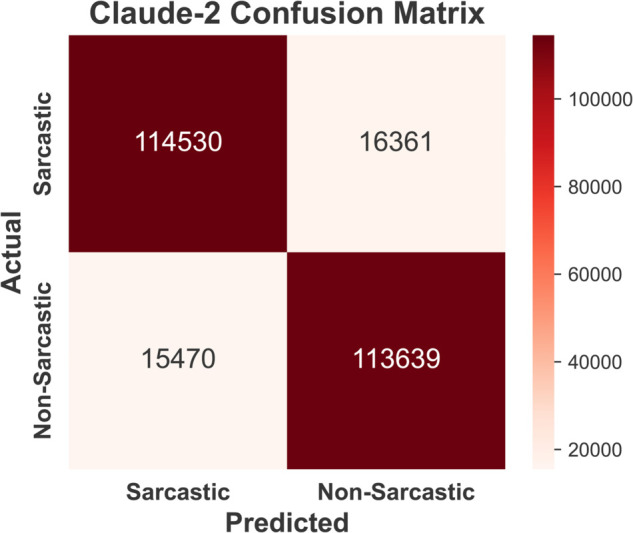
Confusion matrix of Claude-2 model illustrating greater false positives and false negatives, reducing detection reliability.

**Fig 12:**
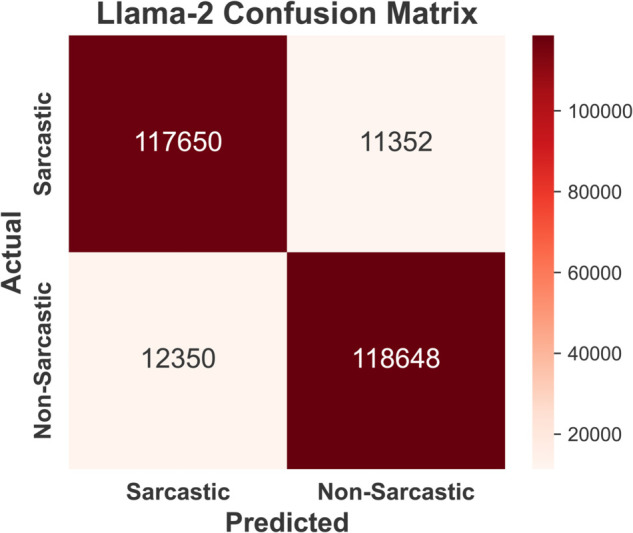
Confusion matrix of Llama-2 model reflecting competitive performance with moderate misclassification rates.

Confusion matrices for sarcasm detection using four models —BERT (Fig 9), GPT-3 (Fig 10), Claude-2 ( Fig 11), and Llama-2 (Fig 12) are provided below. BERT demonstrates exceptional accuracy, particularly in minimizing false negatives (10790) and false positives (10648), indicating its high precision and recall for both classes. GPT-3 exhibits a relatively balanced performance but suffers from higher false negatives (12870) and false positives (14625) compared to BERT, suggesting a slightly reduced sensitivity to sarcasm. Claude-2 and Llama-2 also exhibit robust performance, but Claude-2 records significantly higher false positives (16,361) and false negatives (15,470), which may indicate a challenge in generalizing sarcasm detection. Conversely, Llama-2 exhibits a balanced performance, with false positives (11352) and false negatives (12350) closer to those of GPT-3. However, it slightly outperforms GPT-3 in accurately detecting sarcasm. Overall, while all models perform well, BERT excels in precision and recall, highlighting its superior capability for sarcasm detection in the Reddit corpus.

**Fig 13:**
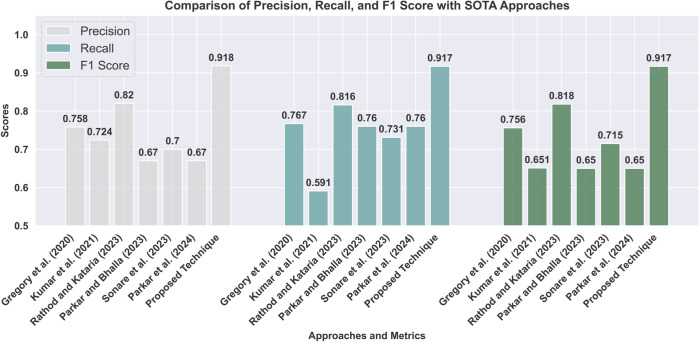
Precision, recall, and F1 score comparison between the proposed method and prior approaches. The fine-tuned BERT with multi-head attention significantly outperforms state-of-the-art baselines.

With an emphasis on essential assessment criteria like Precision, Recall, and F1 Score, Table 9 compares various sarcasm detection methods thoroughly. Gregory *et al*. [38], one of the renowned research projects cited, achieved a Precision of 0.758, Recall of 0.767, and F1 Score of 0.756. This approach lacks multi-head attention and advanced fine-tuning, resulting in lower precision and recall. Kumar *et al*. [39] achieved a recall of 0.591, F1 Score of 0.651, and Precision of 0.724. This work employs simpler embeddings for feature capture, which results in a poorer contextual understanding of sarcasm. With Precision, Recall, and F1 Scores of 0.820, 0.816, and 0.818, respectively, Rathod *et al*. [59] showed robust performance. This approach is limited by the inability to model long-range dependencies, which is inherent to the CNN and LSTM architectures. Parkar *et al*. [60] attained an F1 Score of 0.650, a Precision of 0.670, and a Recall of 0.760. This approach is Over-reliant on Word2Vec embeddings, which lack contextual richness. According to Sonare *et al*., [61], the F1 Score was 0.715, the Precision was 0.700, and the Recall was 0.731. This work struggles with longer sequence lengths and noise in social media data. That being said, the BERT + Multi-Head Attention Mechanism outperformed the rest with a remarkable F1 Score (0.917), Precision (0.918), and Recall (0.917). These outcomes demonstrate the significant improvement of the suggested model over current approaches in detecting sarcasm. The comparative performance of BERT + Multi-Head Attention against state-of-the-art methods is shown in Fig 13.

**Table 9:** Comparison of approaches(SARC dataset).

Approaches	Precision	Recall	F1 Score
Gregory *et al*. (2020) [38]	0.758	0.767	0.756
Kumar *et al*.(2021) [39]	0.724	0.591	0.651
Rathod *et al*. (2023) [59]	0.820	0.816	0.818
Sonare *et al*. (2023) [61]	0.700	0.731	0.715
Parkar *et al*. (2024) [60]	0.670	0.760	0.650
BERT + Multi-Head Attention	**0.918**	**0.917**	**0.917**

The superior performance of our proposed BERT-based model with Multi-Head Attention over the approaches can be attributed to several critical advancements. First, while previous studies relied on traditional embeddings, such as Word2Vec and GloVe, or utilized default transformer configurations, our approach fine-tunes BERT with sixteen multi-head attention mechanisms, extended sequence lengths (128 tokens), and optimized hyperparameters. This ensures the capture of intricate contextual and semantic dependencies specific to sarcasm detection. Second, including an MT step addresses the multilingual nature of social media data, ensuring consistency and reducing noise, which is often neglected in previous works. Finally, our preprocessing pipeline removes redundancies and normalizes text, which, combined with BERT’s bidirectional context-aware embeddings, enables more robust sarcasm detection. These enhancements collectively overcome the limitations of the referenced methods, which either lacked advanced contextual learning capabilities or did not adequately handle noisy and multilingual datasets, as evidenced by their lower F1 scores, precision, and recall metrics.

### 4.3 Ablation study

In this study, we conduct an ablation analysis to assess the contributions of Machine Translation (MT) and Without Machine Translation (WMT), to assess the performance impact of translating sarcastic comments on detection models. We evaluate each model under different configurations, and the results from the ablation study are summarized in Table 10.

**Table 10:** Ablation study for sarcasm detection models.

Model	Configuration	Precision	Recall	F1 Score	Accuracy
BERT	**With Machine Translation**	**0.918**	**0.917**	**0.917**	**0.915**
	Without Machine Translation	0.906	0.905	0.905	0.904
GPT-3	With Machine Translation	0.887	0.901	0.895	0.894
	Without Machine Translation	0.870	0.885	0.876	0.872
Claude-2	With Machine Translation	0.875	0.881	0.878	0.877
	Without Machine Translation	0.855	0.860	0.857	0.856
*LLaMA-2	With Machine Translation	0.912	0.905	0.908	0.908
	Without Machine Translation	0.895	0.900	0.898	0.892

The ablation results demonstrate the value of applying MT to non-English sarcastic comments before classification. Across all models, configurations using MT consistently outperformed their counterparts, WMT. The performance improvements were most notable in the BERT model, which achieved gains of 0.012 in precision and 0.011 in F1 score when MT was applied. These results suggest that translating multilingual inputs into English enhances semantic consistency and improves the model’s ability to understand and detect sarcasm accurately. This is especially relevant when working with datasets like SARC, which include diverse linguistic patterns and idiomatic expressions that may not be equally understood across languages. In summary, Machine Translation proves to be a valuable preprocessing step in sarcasm detection pipelines. Its consistent impact across multiple architectures highlights its potential as a generalizable strategy for improving multilingual sarcasm classification.

## 5 Discussion

### 5.1 Quantitative evaluation of models

This study systematically evaluated various LLMs for sarcasm detection, employing multiple techniques rather than relying on a single approach. Initial experiments involved directly inputting sarcastic text into models such as BERT, GPT-3, Claude-2, and Llama-2, which yielded only modest performance, with precision, recall, and F1 scores ranging from 0.50 to 0.60. Machine Translation (MT) was then applied to standardize multilingual inputs into English, yielding slight improvements across all models. Notably, BERT and GPT-3 demonstrated enhanced performance after MT, achieving scores of up to 0.67 on evaluation metrics, whereas Claude-2 and Llama-2 ranged between 0.52 and 0.57. To further improve results, we explored various embedding techniques, including Word2Vec, fastText, and Paragram, to capture semantic and subword relationships, as well as BERT embeddings for contextual understanding. These embeddings notably improved model performance, with BERT, GPT-3, and Llama-2 reaching between 0.73 and 0.85 on all metrics (see Table 8).

Subsequent fine-tuning of the models, excluding Claude-2 due to its closed configuration, yielded further gains. For BERT, we introduced architectural enhancements, including increasing the number of attention heads from 12 to 16 and transformer blocks from 12 to 16, as well as the sequence length from 128 to 512, to accommodate longer sarcastic expressions. The learning rate was also adjusted to 3*e*^−5^, resulting in a significant boost in predictive accuracy. GPT-3 and Llama-2 were also fine-tuned, achieving their best results using optimized hyperparameters detailed in Tables 5 and 6. Among all models, the fine-tuned BERT emerged as the top performer, effectively leveraging its multi-head attention mechanism to detect nuanced sarcastic cues in context. In contrast, GPT-3 occasionally struggled with subtle irony, while Claude-2 showed the least improvement due to tuning limitations. Llama-2 exhibited strong performance and balanced precision-recall tradeoffs, confirming its robustness in handling complex sarcastic expressions.

### 5.2 Broader theoretical and practical implications

This study contributes to the growing body of research examining how NLP models interact with figurative and pragmatic language phenomena, including sarcasm. For example, Ghosh *et al*. [24] and Gregory *et al*. [38] emphasized the importance of pragmatic incongruity and contextual embeddings in understanding sarcasm. Our study builds on these foundations by demonstrating that a fine-tuned BERT model with optimized attention heads significantly improves sensitivity to these nuanced cues. Unlike traditional methods that rely on surface-level sentiment reversal or lexical surprise, our model empirically confirms that deeper contextual modeling, achieved through multi-head attention and bidirectional encoding, aligns with these theories and offers practical advancements in sarcasm detection.

The effectiveness of the proposed model provides both theoretical and practical insights into sarcasm as a linguistic and psychological phenomenon. Sarcasm often involves a contradiction between literal and intended meaning, aligning with pragmatic theories such as Grice’s Maxim of Quality [62], which is intentionally violated to convey irony. The model’s ability to capture these nuances suggests that sarcasm is fundamentally context-dependent and benefits from advanced representations of long-range semantic dependencies, as supported by research on contextual embeddings [23,33]. From a cognitive psychology perspective, sarcasm requires Theory of Mind or mental state inference [63], where the listener must infer the speaker’s intent through indirect cues. This process effectively fine-tunes attention mechanisms in BERT by attending to multiple semantic layers in text [40,42].

Beyond theoretical contributions, improved sarcasm detection has meaningful applications in real-world scenarios. In marketing and brand management, it helps prevent the misinterpretation of sarcastic reviews [64], preserves analytical integrity, and protects brand reputation. Sarcasm-aware chatbots in customer service can enhance user interactions by responding more empathetically [65]. At the same time, UX designers can leverage sarcasm detection to mitigate the spread of hostile or ironic content on platforms [66]. Additionally, behavioral researchers and psychologists can utilize sarcasm-labeled datasets to analyze humor, digital expression, and social cognition in online communication [67]. These interdisciplinary implications reinforce the broader societal and academic relevance of our proposed framework.

### 5.3 Evaluating models for real-time scenarios

To evaluate the practicality of the proposed model in real-time scenarios, we analyzed its inference speed (IS), memory usage (MU), Feasibility on Edge Devices (FED), and Production Suitability (PS). The proposed model processes an average of 40 comments per second on a GPU-enabled environment (NVIDIA RTX 2060 Super). In comparison, GPT-3 processes 25 comments per second, Claude-2 20 comments per second, and Llama-2 40 comments per second, highlighting BERT’s superior balance of speed and accuracy. While BERT’s memory usage during inference is approximately 10 GB, lighter versions could be explored for resource-constrained devices. Table 11 summarizes the comparison of inference speed, memory usage, and feasibility across the models. These results affirm BERT’s suitability for production environments, though future work should focus on reducing memory usage and further training on diverse sarcastic expressions to improve structural generalization.

**Table 11:** Comparative analysis of inference speed and resource consumption across models.

Model	IS	MU	FED	PS
	(comments/sec)	(GB)		
BERT	40	10	Moderate	High
GPT-3	25	20	Low	Moderate
Claude-2	20	18	Low	Moderate
Llama-2	40	15	Moderate	Moderate

### 5.4 Error analysis and model performance evaluation

We conducted a detailed error analysis to understand the model’s performance better, focusing on the false positives and negatives of each model. BERT outperformed other models, minimizing false positives (299) and false negatives (450), thanks to its bidirectional attention mechanism that effectively captures subtle contextual cues. However, models like GPT-3 and Claude-2 exhibited significantly higher false positives (GPT-3: 16,874; Claude-2: 18,878) and false negatives (GPT-3: 14,850; Claude-2: 17,850), indicating difficulties in detecting sarcasm, particularly in more complex or ambiguous contexts. Llama-2 showed a more balanced performance but still lagged behind BERT in accuracy, with false positives (13,098) and false negatives (14,250) remaining higher than desired.

**Fig 14:**
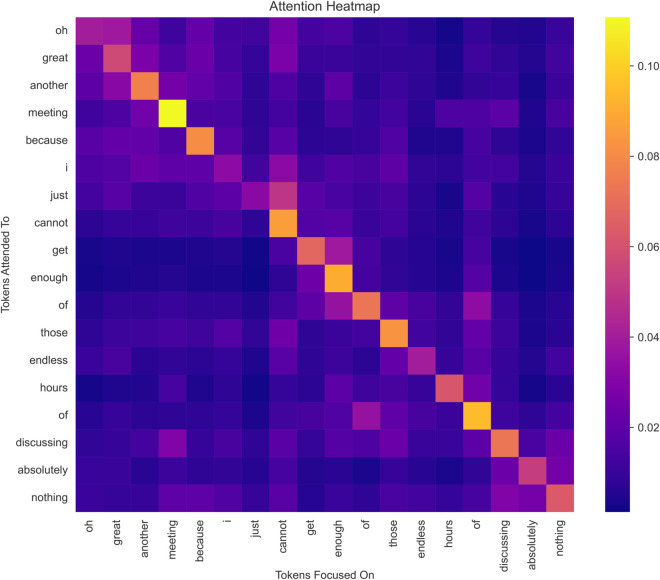
Attention heatmap from the fine-tuned BERT model, showing how multi-head attention highlights key tokens in a sarcastic sentence (e.g., ‘oh great,’ ‘another meeting,’ ‘endless hours’). The visualization demonstrates the model’s ability to focus on lexical cues and contextual dependencies that signal sarcasm, providing interpretability into how the model distinguishes sarcastic from non-sarcastic text.

The errors primarily stemmed from each model’s inability to capture the context-dependent and nuanced nature of sarcasm fully. While BERT excelled due to its fine-tuned architecture, including extended sequence lengths and multi-head attention, GPT-3 and Claude-2 struggled with sarcastic expressions that relied on conflicting sentiments or indirect cues. These challenges underscore the need for task-specific fine-tuning and additional enhancements in contextual embeddings to minimize misclassification. To assess the model’s attention to each word, we evaluate it through an attention heatmap (Fig 14), which clearly shows how much attention is being paid to each word.

### 5.5 Statistical validation of results

We performed several statistical analyses to ensure the robustness and reliability of the observed differences in model performance. First, paired t-tests were conducted to assess the statistical significance of differences in precision, recall, F1 scores, and accuracy between the fine-tuned models (BERT, GPT-3, Claude-2, and Llama-2). The p-values for all key comparisons were less than 0.05, indicating statistically significant differences in model performance.

Additionally, 95% confidence intervals for each performance metric were computed to quantify the precision of our results. The narrow confidence intervals for each metric confirmed the reliability of the performance estimates. For example, the 95% CI for BERT’s F1 score ranged from 0.916 to 0.918, indicating high confidence in its superior performance.

Finally, Cohen’s D effect size was calculated for each model comparison to quantify the magnitude of performance differences. The effect size for comparing BERT and GPT-3 was 1.4, indicating a large and meaningful difference. These statistical analyses prove that the observed differences in model performance are statistically significant and practically substantial.

## 6 Conclusion and future work

This study presents a robust framework for sarcasm detection on social media, introducing a customized BERT model enhanced with multi-head attention, machine translation preprocessing, and task-specific fine-tuning. Experimental evaluations using the SARC dataset and benchmark comparisons against GPT-3, Claude-2, and Llama-2 confirm that our model consistently outperforms these state-of-the-art LLMs across accuracy, precision, recall, and F1 score. The findings highlight the significance of attention optimization and contextual embeddings in transformer-based models, demonstrating that tailored adaptations are more effective than general-purpose architectures for capturing the nuanced semantics of sarcasm. This work not only contributes to methodological advancements in sarcasm detection but also offers practical value for real-world applications such as social media moderation, sentiment-aware customer service bots, and behavioral analysis systems. Furthermore, the integration of machine translation shows the model’s potential for scalable, cross-lingual deployment.

Despite its strengths, the study has a few limitations. Automatic translation can introduce semantic noise in low-resource languages, which can hinder the accuracy of sarcasm recognition. Moreover, sarcasm is often culturally rooted and pragmatically nuanced, relying on idioms, tonal shifts, and context-specific cues that are not always preserved through automatic translation. Prior linguistic research ([64,68] suggests that irony and sarcasm are highly context-dependent and may lose their intended effect when translated across languages. While our ablation studies showed modest improvements using MT, we recognize that future research should empirically assess the fidelity of sarcasm preservation in translated content. Additionally, the model is trained solely on textual input and does not consider multimodal cues, such as emojis, images, or speech tone, which are often crucial for detecting sarcasm in online communication. Future research could explore the integration of such multimodal features and investigate the use of few-shot learning with newer models, such as DeBERTa-v3 or XLM-RoBERTa, to enhance performance in low-data scenarios. Exploring sarcasm-aware conversational agents and human-in-the-loop systems may also open up exciting directions for adaptive, empathetic AI interactions. These pathways offer rich opportunities further to advance the theoretical and practical landscape of sarcasm detection.
